# The importance of a valid assessment of salt intake in individuals and populations. A scientific statement of the British and Irish Hypertension Society

**DOI:** 10.1038/s41371-019-0203-1

**Published:** 2019-04-26

**Authors:** Francesco P. Cappuccio, Peter S. Sever, on behalf of the British and Irish Hypertension Society

**Affiliations:** 1 University of Warwick, WHO Collaborating Centre for Nutrition, Warwick Medical School, Coventry, UK; 2 University Hospitals Coventry & Warwickshire NHS Trust, Coventry, UK; 3 Imperial College London, National Heart & Lung Institute, London, UK

**Keywords:** Diseases, Risk factors

## Introduction

High salt (salt is sodium chloride – 2.5 g of salt contain 1 g of sodium) intake is a major determinant of blood pressure (BP) in individuals and populations [1]. A reduction of salt intake leads to a reduction in BP and is associated with a reduction in the incidence of cardiovascular disease (CVD) [1–3]. However, in the past few years, some epidemiological studies suggested the presence of a J-shaped association between salt (sodium) consumption and CVD [4–9]. These results sparked both scientific and media interest and opened a debate on the wisdom of pursuing population-wide salt reduction policies to reduce CVD, as currently recommended by most national and international health organizations, including the World Health Organization (WHO) [10]. Systematic appraisal of these studies identified a variety of pitfalls, suggesting that their results were based on flawed methodologies [11, 12]. The present scientific statement aims to briefly discuss only one such flaw, the use of biased methods of assessing salt consumption, and the consequences of using such biased estimates of exposure (salt intake) when assessing both individual salt intake (for associations with CVD) and population salt consumption (to evaluate population salt reduction programmes).

## Assessment of salt intake

**Fig. 1:**
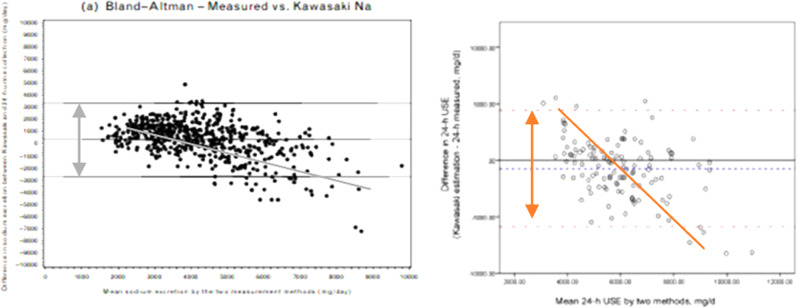
Validation and comparison of the Kawasaki formula to estimate 24 h urinary sodium excretion from a single morning spot urine sample in the PURE Study. On the left it is the validation in 1083 participants from 11 countries^†^ and on the right it is the validation in 120 participants from the Shanxi Province of China^#^. ^†^1083 consecutive individuals attending follow-up clinics over a period of 2–6 months; 87 from India, 153 from China and Colombia, 412 from Argentina, Brazil, Malaysia, South Africa, Turkey, 431 from Canada, Sweden, UAE. ^#^120 participants (60 rural and 60 urban) attending either 3-year or 6-year follow-up visit. Re-drawn from Mente A, et al. J Hypertens. 2014;32:1005–15 (left) and Peng Y, et al. PLoS One 2016;11(2):e0149655 (right)

The assessment of salt intake is fraught with difficulties. Nutritional tools based on either questionnaires or food diaries are inadequate to characterise salt consumption of individuals and populations when compared with biomarkers, and are not recommended as research tools for this purpose [13, 14]. The 24 h urinary excretion of sodium is considered the reference method to assess salt consumption, since ~93% of the sodium ingested, mostly as salt (salt is sodium chloride – 2.5 g of salt contain 1 g of sodium), is eliminated by the kidney in the next 24 h [15]. If we ate the same amount of salt every day, a single 24 h urine collection would indicate with a high degree of precision how much salt we eat. However, due to the high variability of salt consumption in an individual between days and some infradian variability in urinary sodium excretion, independent of intake, many more collections on different days would be required to characterize the habitual individual’s salt consumption [16]. Therefore multiple assessments are needed, in prospective studies, to obtain a reliable estimate of the degree of association between habitual salt consumption and future risk of CVD [3, 17–19]. To overcome the high methodological burden of collecting complete 24 h urine samples in large population-based studies in some settings, then, alternative easier methods have been proposed. Amongst those alternatives the use of ‘spot’ urine collections and the application of different formulae (e.g. Kawasaki, Tanaka, INTERSALT) to derive 24 h urinary sodium excretion have become of popular use [4–9]. The commonest is the Kawasaki [20]. This formula relies on urinary creatinine concentration from a spot collection and 24 h urinary creatinine excretion predicted from age, sex, height, and weight. It is, however, an inappropriate method for estimating salt intake in individuals, due to its unreliability and systematic bias [18]. These extrapolations consistently overestimate at lower levels of salt intake and underestimate at higher levels, introducing a systematic bias, detected in all validation studies performed to date, including the PURE Study [6–9, 12] (Fig. 1).

## Consequences of estimating ‘individual’ sodium excretion in associations studies between salt intake and CVD

**Fig. 2:**
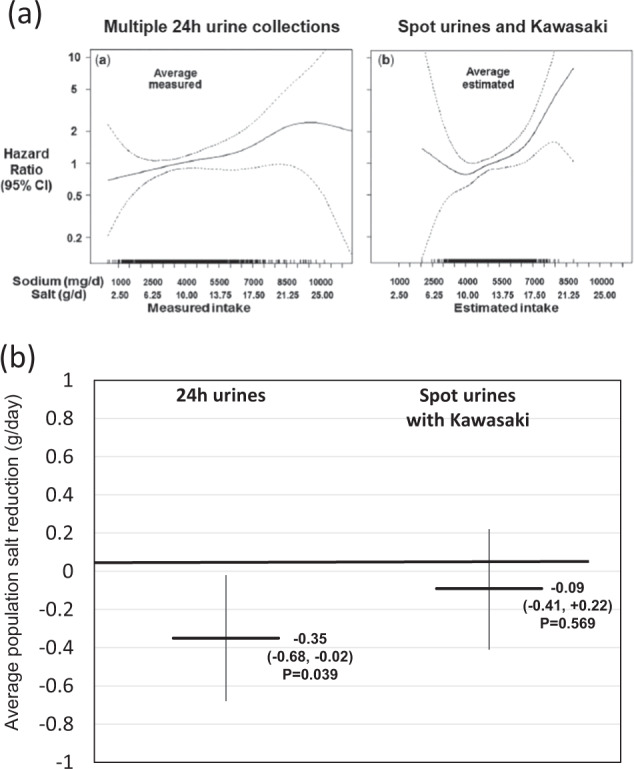
**a** Errors in estimating usual sodium intake by the Kawasaki formula alter its relationship with mortality. Head-to-head comparison with 24 h urinary sodium excretion. Re-drawn from He FJ, et al. Int J Epidemiol. 2018;47:1784–95. **b** Spot urine collections with Kawasaki equation is inadequate to monitor changes in population salt reduction programmes. Head-to-head comparison with 24 h urinary sodium excretion. Drawn from Huang L, et al. Int J Epidemiol 2018;47:1811–20

An accurate and unbiased measurement of ‘individual’ dietary sodium consumption is paramount in aetiological epidemiology. In prospective studies, where multiple complete 24 h urinary sodium collections have been used to measure exposure to salt consumption, consistent and graded relationships have been described between sodium excretion and health outcomes (CVD and all-cause mortality) in general populations, as well as in patients groups [3, 17–19]. On the contrary, when studies have used the Kawasaki formula, a J-shaped relationship has been obtained [4–9]. Finally, when a head-to-head comparison was carried out between measures of salt consumption obtained from repeated 24 h urine collections compared to a spot urine, in the prospective assessment of salt and mortality, there was a graded relationship when salt consumption was assessed with multiple 24 h collections (with no evidence of increased risk at lower levels up to 3 g of salt per day) whereas an ‘erroneous’ J-shaped curve was generated when using the Kawasaki formula from spot urines [21] (Fig. 2a). Therefore ‘spot’ urine collections with the use of the Kawasaki formula are an inappropriate method for studying associations in individuals in prospective studies, and should not be used in the context of prospective assessment of salt consumption as a predictor of health outcomes.

## Consequences of estimating ‘population’ average salt consumption in the evaluation of salt reduction programmes

An accurate and unbiased measurement of ‘average’ dietary sodium consumption in population groups is paramount in public health and policy. The knowledge of a reliable estimate of population intake will help public health professionals in several ways. First, it will establish the size of the problem. (How much salt does my population eat?) Second, it will provide the gap from set targets. (How much do I have to reduce the average salt consumption to achieve WHO targets of 5 g per day?) Third, it will help evaluate the intervention in populations by determining changes in average intake over time. Fourth, it will inform health economic evaluations of health impact and motivate continuous political commitment. So, the choice of the right method for measuring salt consumption is equally important in this setting. In a recent study in South Africa, the validity of different formulas—including Kawasaki—applied to spot urine estimates of sodium were tested against 24 h urinary sodium measurements. The study showed that these formulas all fall short of an ideal scenario when assessing the presence and size of the bias [22]. In the case of the Kawasaki formula the size of the bias was equivalent to 5.6 g of salt [22]. The important implication of these results for policy is that all these formulas introduce a bias with large inaccuracy both in the baseline estimation and, more importantly, they do not enable them to detect smaller changes in population salt consumption over time ensuing from salt reduction programmes. For instance, if spot urines with Kawasaki estimates had been applied to the evaluation of the 8-year UK national salt reduction programme, a detection of 1.4 g per day reduction achieved over that period would not have been easy to detect given the presence of a bias three times as large. Therefore, the effectiveness of the population intervention would have been missed, with crucial implications for further investments and commitments towards that public health [23]. The real possibility of this scenario has been reported in the recent head-to-head comparison of an evaluation of the effectiveness of a 6-to-24 months salt-substitution programme in China within the framework of a well-controlled randomised clinical trial [24]. Over the time of intervention there was a statistically significant reduction in average sodium consumption of 0.35 g per day (*p* = 0.039) when assessed by 24 h urinary sodium excretion. However, when spot urines with Kawasaki equation were used, the change was detected as −0.09 g per day (*p* = 0.569), a quarter of the real effect (Fig. 2b). Therefore ‘spot’ urine collections with the use of the Kawasaki formula are an inappropriate method for studying population chages and should not be used in the context of a public health evaluation of the effectiveness of salt reduction programmes.

## Conclusions

The evidence supporting global actions for a moderate reduction in salt consumption to prevent CVD is strong and new controversial studies, based on flawed methodology, are inappropriate to address the complex associations between salt intake and CVD outcomes and the evaluation of population salt reduction programmes. They should not overturn the ongoing concerted public health action to reduce salt intake globally.
