# Correction: The importance of a valid assessment of salt intake in individuals and populations. A scientific statement of the British and Irish Hypertension Society

**DOI:** 10.1038/s41371-020-0317-5

**Published:** 2020-02-26

**Authors:** Francesco P. Cappuccio, Peter S. Sever, on behalf of the British and Irish Hypertension Society

**Affiliations:** 1 University of Warwick, WHO Collaborating Centre for Nutrition, Warwick Medical School, Coventry, UK; 2 University Hospitals Coventry & Warwickshire NHS Trust, Coventry, UK; 3 Imperial College London, National Heart & Lung Institute, London, UK

**Keywords:** Diseases, Risk factors

**Correction to: Journal of Human Hypertension**

10.1038/s41371-019-0203-1

This article was originally published under Nature Research’s License to Publish, but has now been made available under a [CC BY 4.0] license. The PDF and HTML versions of the article have been modified accordingly.

